# Epidemiological analysis of injury in Shandong Province, China

**DOI:** 10.1186/1471-2458-8-122

**Published:** 2008-04-17

**Authors:** Jixiang Ma, Xiaolei Guo, Aiqiang Xu, Jiyu Zhang, Chongqi Jia

**Affiliations:** 1 Department of Non-Communicable Disease Prevention, Shandong Centre for Disease Control and Prevention, 72 Jingshi Road, Jinan 250014, China; 2 Department of Epidemiology and Health Statistics, Shandong University, China

## Abstract

**Background:**

Injury is an emerging public health problem with social development and modernization in developing countries. To describe the prevalence and burden of injury and provide elaborate information for policy development, we conducted a community-based household survey in the Shandong Province of China.

**Methods:**

The survey was conducted in 2004. Participants were selected by a multi-stage random sampling method. Information on injuries occurring in 2003 was collected in four cities and six rural counties in Shandong Province, China.

**Results:**

The estimated incidence rate of injury in Shandong Province was 67.7 per 1,000. Injury incidence was higher in rural areas (84.3 per 1,000) than in urban areas (42.9 per 1,000), and was higher among males (81.1 per 1,000) than females (54.1 per 1,000).

The average years of potential life lost is 37.7 years for each fatal injury. All injuries together caused 6,080,407 RMB yuan of direct and indirect economic loss, with traffic injuries accounting for 44.8% of the total economic loss.

**Conclusion:**

Injury incidence was higher among males than females, and in rural areas than in urban areas. Youngsters suffered the highest incidence of injury. Injury also caused large losses in terms of both economics and life, with traffic injuries contributing the most to this loss. Strategies for prevention of injury should be developed.

## Background

An estimated 5 million people worldwide died from injuries in 2000, with a mortality rate of 83.7/100,000 population, accounting for 9% of the world's deaths in 2000 and 12% of the world's burden of disease[1].

In China, injuries accounted for approximately 750,000 deaths and 3.5 million hospitalizations in 1999, acting as the leading cause of death for those aged 1 to 44 years[2]. The estimated annual economic cost of injury is equivalent to 12.5 billion US dollars, almost four times the total public health services budget of China[3]. Injury has become an important public health problem in China[4].

Shandong Province, located in the eastern part of China, has an area of 156,700 km^2 ^and a population of about 90 million. According to vital statistics, the injury fatality rate was 57.02 per 100,000 in Shandong Province in 2001, accounting for 9.08% of all deaths[5].

We conducted a community-based household survey in the Shandong Province of China in order to describe the pattern of injury occurrence, understand the circumstances and risk factors associated with injury, and illustrate the burden caused by injury.

## Methods

Participants were selected using a stratified, multi-stage random sampling method. According to national socioeconomic classification criteria, an urban area was classified as a large, middle size, or small city based on population size. Rural areas were classified as rural area class 1, rural area class 2, and rural area class 3 based on the health and economic situation in the area. Two city districts in each urban area level, three counties in each rural area level, two sub-districts (towns) in each district (county), three resident commissions (villages) in each sub-district (town), and 200 households in each resident commission (village) were selected randomly. All permanent residents (inhabitancy of > 6 months) in selected households were to be interviewed.

The survey was carried out in four cities and six rural counties from April to June in 2004. Interviewers from local disease control institutions or hospitals underwent standard training. The study was approved by the Ethics Committee of the Shandong Centre for Disease Control and Prevention. After the interviewers described the study to each family in the sample, informed verbal consent was sought.

All members of the chosen households were interviewed. Information was obtained from relatives if an interviewee was unavailable or younger than ten years. The survey focused on injuries that occurred in 2003 and met any of the following three criteria: 1) injuries diagnosed in medical institutions; 2) injuries requiring treatment or care; and 3) injuries that required rest for at least half a day. In the case of multiple injuries, all episodes were included. Injuries were classified according to ICD-10.

### Main indexes

The incidence rate of injury by individual was calculated. Average household income in 2003 was surveyed to measure the economic status of respondents. Years of potential life lost (YPLL), working years of potential life lost (WYPLL), valued years of potential life lost(VYPLL)[6], and direct and indirect economic loss (IEL) caused by injury were analyzed (Table 1).

**Table 1:** Health economic indexes and calculation*

**Abbreviation**	**Name**	**Formula and calculation**
YPLL	Years of potential life lost	$YPLL=\sum_{i=1}^{N-1}d_{i}(N-i)$
WYPLL	Working years of potential life lost	$WYPLL=\sum_{i=1}^{W-1}d_{i}(N-W)+\sum_{i=w}^{N}d_{i}(N-i)$
VYPLL**	Valued years of potential life lost	$VYPLL=\sum_{i=1}^{N-1}\left[\left(I_{1}+C_{1}-P_{1})+(P_{0}-I_{0}-C_{0}\right)\right]$
IEL***	Indirect economic loss	$IEL=\sum_{i=W}^{N-1}\left(T_{L}I_{A}\right)$

Variables: i=age at death; di=number of deaths at age i; N= upper cut-off age, 70 was used here; W= lower cut-off age of working, 20 was used here; I_1 _= invested years; Io = uninvested years; P_1 _= produced years; P_0 _= unproduced years; C_1 _= consumed years; C_0 _= un-consumed years; T_L _= Lost Time caused by injury (years for fatal injury and days for nonfatal injury; I_A _= Average income per lost time (per year for fatal injury and per day for nonfatal injury)

* Cited from [6].

** Lifetime is divided into three segments, as investment (0–19), producer (20–59), and consumer (60–70). Invested years, produced years and consumed years were years one have been invested during investment period, years one have made contribution during producer period and years one have consumed during consumer period.

*** Indirect economic loss is classified into indirect economic loss 1(IEL1) and indirect economic loss 2 (IEL2), which is the value of lost labour caused by nonfatal and fatal injuries, respectively. It was calculated according to loss of work days/years caused by injury and per capita income of per day/year. Per capita income was average social dominatable income for urban dwellers and average pure earnings for rural dwellers according to local statistics in 2003.

### Data analysis

Epidata 3.0 and Stata 8.0 were used for data input and analysis. Rates and ratios were the main indexes of the study. Bivariate analyses were performed using cross tabulations. A chi-squared test was used to test for homogeneity. Multivariate logistic regression analysis was also used to identify risk factors of injury incidence.

## Results

A total of 24,438 participants of all ages completed the survey. No significant differences were found on the age and sex distribution between the sample and population (Table 2). The response rate of the survey was 97.75%. In the survey, 1,655 respondents reported suffering an injury, resulting in an incidence of 67.7 per 1,000 per year. Among the injuries reported, 17 cases were fatal, leading to a fatality rate of 69.56/100,000.

**Table 2:** Demographic composition of the sample and population

Variables	Population (%)	Sample (%)
Gender		
Male	50.6	50.4
Female	49.4	49.6
	X^2 ^= 0.47, P > 0.05	
Age group		
0-	5.1	3.9
5-	15.7	10.2
15-	50.3	50.1
45-	17.3	22.0
60+	11.6	13.8
	X^2 ^= 2.29, P > 0.05	

### Distribution of injury incidence

Injury incidence broken down by demographic factors and mechanisms is shown in Table 3. The incidence of total injury was higher for males (81.1 per 1,000) than females (54.1 per 1,000) (X^2 ^= 70.6, P < 0.01). Injuries for males predominated at all ages, except for those over age 60. Males were characterized by a high incidence of traffic injuries (17.5 per 1,000) and females by a high incidence of falls (12.3 per 1,000). The fatality rate for males (105.57/100,000) was also higher than for females (32.99/100,000) (X^2 ^= 72.4, P < 0.01).

**Table 3:** Demographic and injury mechanism distribution of individuals suffered injury (N/1 000)*

**Factors**	**Age group**					
	
	0-	5-	15-	45-	60+	Total
Sex						
Male	39(69.3)	140(109.1)	497(80.9)	216(81.0)	107(64.6)	999(81.1)
Female	20(40.7)	94(78.1)	237(39.2)	171(63.2)	134(79.9)	656(54.1)
						X^2 ^= 70.6, P < 0.01
Area						
Urban	11(28.5)	45(53.2)	206(40.5)	92(45.0)	66(46.5)	420(42.9)
Rural	48(71.9)	189(115.2)	528(74.4)	295(88.6)	175(91.4)	1235(84.3)
						X^2 ^= 158.9, P < 0.01
Mechanism						
Traffic accident	3(2.8)	24(9.7)	182(14.9)	72(13.4)	30(9.0)	311(12.7)
Fall	19(18.0)	46(18.5)	82(6.7)	78(14.5)	92(27.6)	317(13.0)
Collision	4(3.8)	47(18.9)	93(7.6)	50(9.3)	17(5.1)	211(8.6)
Strain	4(3.8)	27(10.9)	131(10.7)	45(8.4)	44(13.2)	251(10.3)
Cut	2(1.9)	21(8.4)	83(6.8)	30(5.6)	8(2.4)	144(5.9)
Squeeze	1(0.9)	13(5.2)	41(3.4)	25(4.7)	7(2.1)	87(3.6)
Burn	10(9.5)	7(2.8)	38(3.1)	23(4.3)	11(3.3)	89(3.6)
Poisoning	1(0.9)	2(0.8)	14(1.1)	19(3.5)	14(4.2)	50(2.0)
Animal bite	14(13.3)	44(17.7)	54(4.4)	37(6.9)	16(4.8)	165(6.8)
Total	59(56.0)	234(94.1)	734(60.2)	387(72.0)	241(72.3)	1,655(67.7)

* Differences between different sexes, areas were examined using Chi-square test.

Age-specific incidence of injury was the highest among males aging 5 to 14 and was the lowest among females aged 15 to 44. A high incidence of falls and collisions was observed in the 5 to 14 age group. Injuries in the 15 to 44 age group accounted for 44.3% of all injuries occurred.

The five leading causes of injury were falls (13.0 per 1,000), traffic injuries (12.7 per 1,000), strains (10.3 per 1,000), collisions/wounds (8.6 per 1,000), and bites by insects or animals (6.8 per 1,000).

Injury incidence by social economic factors is shown in Table 4. There are significant differences of injury incidence among different educational levels (X^2 ^= 85.8, P < 0.01), with lower injury incidence among those with higher educational attainment. Injuries were more frequently observed among those who were illiterate (99.4 per 1,000) compared to others.

**Table 4:** Social economic composition of individuals suffered injury (N/1,000)*

**Factors**	**Sex**		
	Male	Female	Total
Education			
Illiterate	181(101.5)	61(93.7)	242(99.4)
Elementary school	168(58.4)	271(98.6)	439(78.0)
Junior high school	170(40.2)	429(88.0)	599(65.8)
Senior high school	88(42.8)	148(61.3)	236(52.8)
College and above	17(31.4)	35(39.9)	52(36.6)
			X^2 ^= 85.8, P < 0.01
Occupation			
Government employee	12(47.8)	17(38.7)	29(42.0)
Labourer	72(40.7)	209(79.5)	281(63.9)
Professional	24(45.2)	66(77.1)	90(64.9)
Farmer	217(59.5)	275(79.0)	492(69.0)
Business and service	32(35.3)	66(89.6)	98(59.6)
Student	129(70.0)	217(104.7)	346(88.4)
Retired	49(67.4)	45(63.3)	94(65.4)
Housework	78(55.5)	22(101.4)	100(61.6)
Unemployed	8(34.8)	16(67.8)	24(51.5)
Others	4(22.5)	13(64.0)	17(44.6)
			X^2 ^= 43.9, P < 0.01
Average income (RMB yuan/year)			
< 2000	304(71.9)	432(101.7)	736(86.8)
2000-	192(48.1)	332(81.5)	524(65.0)
5000+	155(43.0)	218(59.0)	373(51.1)
			X^2 ^= 80.8, P < 0.01

* Differences among classifications of education, occupation, average income were examined using Chi-square test.

A difference in injury incidence by occupation was observed (X^2 ^= 43.9, P < 0.01). Students (8.8%), farmers (6.9%), retired individuals (6.5), professionals (6.5%) and labourers (6.4%) show a high incidence of injury, accounting for 82.9% of total injuries. Government employees were most likely to be affected by traffic injuries, followed by labourers and those in the business and service industry, while the retired and preschool children were mainly affected by falls.

Injury incidence also varied (X^2 ^= 80.8, P < 0.01) among different levels of income. Respondents with lower income tended to have a higher incidence of injury. Among those with the lowest income (2,000 yuan or below), incidence of a fall was observed most frequently. Sprain was the leading mechanism among those with the highest income (5,000 yuan or above).

Injury incidence in rural areas (84.3 per 1,000) was higher than in urban areas (42.9 per 1,000) (X^2 ^= 158.9, P < 0.01), with the majority of injuries occurring in rural areas (74.6%).

Logistic regression analysis shows that sex, area, education, income, and occupation are significantly associated with injury incidence (Table 5). Males are 60% more likely to incur an injury compared with females. Rural residents have an injury incidence rate 1.99 times that of urban residents. Analysis by level of education shows a tendency for those with higher education levels to have a lower risk of injury incidence. The odds of injury for the illiterate group are 3.17 times that of the college and above group. Among different income levels, those with the lowest income (< 2,000) show the highest risk of injury incidence, while the middle level (2,000–4,999) show the lowest risk. Analysis of occupation shows that farmers have the lowest risk of injury incidence, while students and professionals face a higher risk.

**Table 5:** Multivariate logistic regression analysis of injury incidence

Indicators	B	SE	P	OR	95.0% C.I. for OR	
					Lower	Upper
**Sex **(Male vs Female)	0.47	0.06	< 0.01	1.60	1.43	1.79
**Area **(Rural vs Urban)	0.69	0.08	< 0.01	1.99	1.69	2.34
**Education**			< 0.01			
Illiterate	1.15	0.18	< 0.01	3.17	2.23	4.51
Elementary school	0.69	0.17	< 0.01	2.00	1.44	2.77
Junior high school	0.53	0.16	< 0.01	1.70	1.23	2.34
Senior high school	0.38	0.17	< 0.01	1.47	1.06	2.03
College and above				1		
**Average Income**						
< 2000	0.23	0.06	< 0.01	1.26	1.12	1.43
2000-				1.00		
5000+	0.16	0.09	> 0.05	1.17	0.99	1.38
**Occupation**						
Farmer				1		
Government employee	0.19	0.21	> 0.05	1.21	0.81	1.82
Labourer	0.40	0.09	< 0.01	1.49	1.26	1.77
Professional	0.55	0.13	< 0.01	1.74	1.34	2.25
Business and service	0.19	0.12	> 0.05	1.21	0.97	1.53
Student	0.61	0.08	< 0.01	1.84	1.57	2.14
Retired	0.42	0.13	< 0.01	1.52	1.18	1.95
Housework	0.13	0.12	> 0.05	1.14	0.90	1.44
Unemployed	0.45	0.22	< 0.05	1.57	1.02	2.43
Others	0.22	0.26	> 0.05	1.24	0.75	2.07

*Goodness of fit: P < 0.05.

### Characteristics of injury occurrence

Injuries occurred mostly at home (31.7%), in a highway/street (31.2%), or at the workplace (22.5%). Injuries at these three places accounted for 85.5% of the total injuries. For males, the injuries were likely to occur in a street or highway, while females were most likely to be injured at home. Injuries in the street/highway generally resulted from traffic injuries (56.3%), while those sustained at home mainly involved falls (26.4%). Injuries at the workplace were diverse, involving collisions (18.3%), strains (17.8%), cuts (16.8%), and falls (16.0%).

Injuries were most likely to happen during work hours or housework. Injuries occurring during work hours mainly involved falls (17.5%), collisions (17.1%), cuts (15.4%), and strains (15.2%). During housework time, incidences of falls (24.7%), strains (19%), and cuts (17.3%) were higher. A high incidence of traffic injuries (57.7%) was observed while shopping and commuting to and from work.

### Severity of injury

Of all injuries, 75.0% were minor and did not need inpatient care, 20.3% were moderate and required hospitalization but did not disable the patient, and 4.7% were serious injuries that left the patient disabled, yielding a disability rate of 3.4 per 1,000 (2.6 per 1,000 for male, 0.8 per 1,000 for female). Disabling injuries were mainly caused by traffic injuries (34.9%), falls (21.7%), and strains (13.4%).

On average, injuries led to 13.6 days (14.9 for male, 11.7 for female) of rest, and 5.4 days (6.1 for male, 4.4 for female) of hospitalization. Explosions, traffic injuries, and falls caused longer periods of both hospitalization and rest.

### Life lost caused by injury

In the survey, 17 persons died of injury, and injuries caused 662 YPLL, 534 WYPLL, and 411 VYPLL. For each fatal injury, the average YPLL, WYPLL, and VYPLL were 38.9, 31.4 and 24.2 mean years, respectively. Traffic injuries and falls were the two major causes of YPLL, leading to 251 and 136 mean years of YPLL, and accounting for 37.9% and 20.5% of the total, respectively.

### Economic loss caused by injury

In the survey, the 1,772 total injuries caused 2,080,156 and 4,000,251 RMB yuan of direct and indirect economic loss, averaging 1,174 and 2,257 RMB yuan for each injury (Table 6). The contribution of males was more than three times that of females. Injuries in rural and urban areas caused economic losses of 3,275,661 and 2,804,746 RMB yuan, respectively. The economic costs of injury in rural areas was slightly higher than that in urban areas. Traffic injuries led to an economic cost of 2,723,713 RMB yuan, accounting for 44.79% of the total.

**Table 6:** Economic loss* caused by injury

**Mechanisms**	**Direct loss**		**Indirect loss 1**		**Indirect loss 2**		**Total**	
	
	RMB Yuan	%	RMB Yuan	%	RMB Yuan	%	RMB Yuan	%
Traffic injury	881235	42.4	146675	35.0	1695802	47.4	2723713	44.8
Fall	496011	23.8	108324	25.9	857164	23.9	1461499	24.0
Struck	179766	8.6	34833	8.3	351619	9.8	566219	9.3
Cut	122883	5.9	26095	6.2	340112	9.5	489090	8.0
Animal bite	23836	1.2	5453	1.3	260249	7.3	289538	4.8
Strain	99517	4.8	44157	10.5			143674	2.4
Burn	83515	4.0	19006	4.5			102520	1.7
Squeeze	71967	3.5	17055	4.1			89021	1.5
Explosion	48501	2.3	5659	1.4			54160	0.9
Poisoning	43264	2.1	7378	1.8			50642	0.8
Others	29661	1.4	4153	1.0	76516	2.1	110330	1.8
Total	2080156	100	418789	100	3581462	100	6080407	100

* Direct loss is the medical cost caused directly by injury accident; Indirect economic loss is classified into indirect economic loss 1(IEL1) and indirect loss 2(IEL2), which is the value of lost labour caused by nonfatal and fatal injuries, respectively.

## Discussion

The survey shows an incidence rate of 66.7 per 1,000 and fatality rate of 69.6/100,000 for total injuries in the Shandong Province. The injury incidence in our study was higher than that of Shijiazhuang (4.32%), Shenzhen (6.35%)[7], and 4 rural communities (6.51%)[8], but was lower than that in four communities in the Zhejiang Province (16.11%) in China. It was also higher than that in Tanzania (3.27%)[9] and Pakistan (4.1%)[10], but lower than that in the 12-month cohort study in India (12.7%)[11] and in the state of Colorado in the United States (14.7%)[12].

The study found a higher injury incidence among males compared with females. Traffic injuries show an extraordinarily higher incidence among males than females, but there is a higher incidence of falls among females in the 60 years and older age group.

A higher incidence of injury was observed in rural areas compared with urban areas. Residents in rural areas are estimated to have a risk of injury incidence that is 1.99 times that in urban areas. This is similar to findings from other studies[9,12,13], but different from the study in Uganda, in which a high incidence of injury in urban areas was found[14]. In contrast to other studies in developing countries, a higher incidence of cuts in rural studies was not found in our study, as in Ghana[13]and Tanzania[9], nor lower incidence of traffic injuries, like Pakistan[10] and Bangladesh[15]. This may be a reflection of the motorization of agriculture activities in rural areas of the Shandong Province.

For different ages, those in the 5 to 14 year-old group and the 60 years and older group had higher injury incidence rates, and the main cause of injury for these two age groups was falling. This is different from two U.S. studies showing a lower injury occurrence among elders[12,16].

Socio-economic status has been documented to be an important determinant of injury. In our study, those with both the lowest and highest income were at a higher risk of injury incidence. However, a study in America showed that income and education in the multivariate models were largely unrelated to overall injury morbidity[17].

Home, highway/street, and workplace were found to be the major sites where most injuries happened. This was different from the study in Bangladesh, in which injuries in a highway/street were not common[15].

Based on the results of this study, we estimate that 6,522,955 cases of injury and 62,585 deaths resulting from injury occurred in Shandong in 2003, and the total number of injuries caused 22.3 billion RMB yuan in economic cost, accounting for 1.8% of the GDP in the Shandong Province in the same year. Traffic injuries accounted for 44.79% of the total economic loss.

### Limitations of the study

The findings of this study are subject to several limitations. First, the information is based on a self-reported survey requiring respondents to recall injuries occurring within the past year, which is subject to recall bias [18-21] and might result in an underestimation of injury occurrence. There is also potential for bias due to unreliable memory or embarrassment regarding certain types of injuries, such as assaults or domestic violence. This may also lead to underestimated injury rates.

In the study, clinical injury severity assessment was not available. Disability days were used instead as a measure of severity of injury. This can only result in a crude evaluation of injury severity.

Logistic regression method was employed to evaluate the risks of injury incidence. The Hosmer-Lemeshow test showed poor fitness of the model, which might attribute to the large size of the study sample.

Despite its limitations, this study has generated information that is useful for targeting prevention at the local level.

## Conclusion

Our study demonstrates that injury has become a public health problem in the Shandong Province of China as the result of social urbanization and modernization. Injuries have caused large losses of life as well as economic losses. Falls and traffic injuries were the two most common causes of injury.

According to the characteristic of injury incidence, prevention priority should be given to traffic injuries, especially in rural areas. Major causes of traffic injury need to be further studied. Collaborations between police, traffic, and health departments are necessary for traffic injury prevention. Adolescents (5–14 years) suffered a higher incidence of falls, collision, and traffic injury. This was also consistent with the high injury incidence among students. Education in schools on corresponding injury prevention should be an effective measure for protection of this population.

## Abbreviations

years of potential life lost (YPLL), working years of potential life lost (WYPLL), valued years of potential life lost (VYPLL), indirect economic loss (IEL)

## Competing interests

The author(s) declare that they have no competing interests.

## Authors' contributions

JM designed and conducted the study, performed statistical analysis, and wrote the initial draft and revisions of the manuscript after consultation with other authors. XG and AX participated in the design, study, and revision of the manuscript. JZ and CJ participated in statistical analysis and coordination, as well as in revision of manuscript. All authors have read and approved the final manuscript.

## Pre-publication history

The pre-publication history for this paper can be accessed here:



## References

[B1] Peden M MGK (2002). The injury chart book: a graphical overview of the global burden of injuries.

[B2] Ministry of Health PRC (2000). 1999 China National Health Statistics..

[B3] Zhou Y, Baker TD, Rao K, Li G (2003). Productivity losses from injury in China. Inj Prev.

[B4] Li GH, Baker SP (1991). A comparison of injury death rates in China and the United States, 1986. Am J Public Health.

[B5] Yu  GF, Guo XL,  Li WK, Yang YH, Ma JX (2004). Analysis of death causes in disease surveillance point system of Shandong Province. Chin J Public Health.

[B6] Gardner JW, Sanborn JS (1990). Years of potential life lost (YPLL)--what does it measure?. Epidemiology.

[B7] Zhang Q, Zhang S, Liang H (2001). Study on the epidemic characteristics and burden of injuries among inhabitants in Shenzhen. Zhonghua Liu Xing Bing Xue Za Zhi.

[B8] Yang GH, Ma JM, Wang LJ (2004). Survey on injury in four rural communities in China. Zhonghua Liu Xing Bing Xue Za Zhi.

[B9] Moshiro C, Heuch I, Astrom AN, Setel P, Hemed Y, Kvale G (2005). Injury morbidity in an urban and a rural area in Tanzania: an epidemiological survey. BMC Public Health.

[B10] Ghaffar A, Hyder AA, Masud TI (2004). The burden of road traffic injuries in developing countries: the 1st national injury survey of Pakistan. Public Health.

[B11] Sathiyasekaran BW (1996). Population-based cohort study of injuries. Injury.

[B12] Leff M, Stallones L, Keefe TJ, Rosenblatt R, Reeds M (2003). Comparison of urban and rural non-fatal injury: the results of a statewide survey. Inj Prev.

[B13] Mock CN, Abantanga F, Cummings P, Koepsell TD (1999). Incidence and outcome of injury in Ghana: a community-based survey. Bull World Health Organ.

[B14] Kobusingye O, Guwatudde D, Lett R (2001). Injury patterns in rural and urban Uganda. Inj Prev.

[B15] Rahman F, Andersson R, Svanstrom L (1998). Health Impact of Injuries: A Population-Based Epidemiological Investigation in a Local Community of Bangladesh. Journal of Safety Research.

[B16] Benson V, Marano MA (1998). Current estimates from the National Health Interview Survey, 1995. Vital Health Stat 10.

[B17] Cubbin C, LeClere FB, Smith GS (2000). Socioeconomic status and the occurrence of fatal and nonfatal injury in the United States. Am J Public Health.

[B18] Moshiro C, Heuch I, Astrom AN, Setel P, Kvale G (2005). Effect of recall on estimation of non-fatal injury rates: a community based study in Tanzania. Inj Prev.

[B19] Mock C, Acheampong F, Adjei S, Koepsell T (1999). The effect of recall on estimation of incidence rates for injury in Ghana. Int J Epidemiol.

[B20] Harel Y, Overpeck MD, Jones DH, Scheidt PC, Bijur PE, Trumble AC, Anderson J (1994). The effects of recall on estimating annual nonfatal injury rates for children and adolescents. Am J Public Health.

[B21] Landen DD, Hendricks S (1995). Effect of recall on reporting of at-work injuries. Public Health Rep.

